# On the Iodides of Quina and Cinchona

**Published:** 1846-01

**Authors:** 


*On the Iodides of Quina and Cinchona.*—Dr. A. T. Thomson has recently succeeded in preparing an iodide of quina, and of cinchona; two compounds which we agree with him in thinking, are likely to form valuable additions to the materia medica, inasmuch as they contain in themselves the combined properties of a most efficient tonic, and one of the most valuable deobstruents which we possess. One of the great objections to the administration of iodine and iodide of potassium, is the production of that derangement of the system denominated *iodism.* Now this is likely to be prevented by the tonic influence of the quina or cinchona. It is true that we already possess such a combination in iodide of iron, but in many instances, where the influence of such conjoint powers is required, preparations of iron cannot be borne.

The *iodide of Quina* is prepared by triturating together, in a mortar, 164-55 grains of the pure quina, and 126-3 grains of iodine; the latter being added to the former until the whole is intimately mixed; and then boiling the mixture in a moderate quantity of distilled water at first, adding more by degrees, until as much is added as will give one grain of the iodide for each fluid drachm of the solution. During the boiling, a deep-brown resinous-like substance is formed, apparently insoluble in water, which subsides to the bottom when the solution cools.

This substance is brittle, tasteless, inodorous, and affords no indication of the presence of either iodine or quina; it is partially soluble in boiling alcohol. Dr. Thomson has not been able to ascertain its nature.

The iodide of quina, in solution, is of a pale straw color, limpid, evolving a faint odor of iodine, and impressing upon the palate the bitter of quina; that it contains no free iodine is evinced by testing it with starch, whilst the existence of the iodine is immediately demonstrated by the developement of the deep indigo-blue color of the iodide of amadine, on adding a drop of nitric acid to the solution containing the starch. The quina in the solution of the iodide is precipitated by the infusion of galls in the form of a tannate; and, in its simple state, when the solution of pure potassa is added to the solution.

*The iodide of Cinchona* is prepared in the same manner as the iodide of quina, taking 156-55 grains of the alkaloid, instead of 164-55. The quantity of brown, resinous-like matter is less than in the preparation of the iodide of quina; but it closely resembles it in its physical characters, its insolubility in water, and its solubility in alcohol. The solution is nearly inodorous, has the bitter taste of the cinchona, and a rather deeper straw-color than the solution of iodide of quina. It is limpid, and answers to the same tests as the iodide of quina.—*Ranking's Half-Yearly Abstract.*

